# Postictal psychosis : Case Report and Literature Review

**DOI:** 10.1192/j.eurpsy.2024.1011

**Published:** 2024-08-27

**Authors:** S. Ajmi, M. Bouhamed, K. Makni, S. Hentati, I. Feki, R. Sallemi, J. Masmoudi

**Affiliations:** Psychiatry A, Hedi Chaker University Hospital, Sfax, Tunisia

## Abstract

**Introduction:**

The prevalence of psychosis in patients with epilepsy is estimated approximately 7.8%. However, postictal psychosis appears to be much less common, with a prevalence of 2% in epilepsy. Postictal psychosis is defined as psychotic episodes starting within less than one week after an epileptic seizure.

**Objectives:**

Our aim was to study the clinical characteristics and the therapeutic options through a case report and a review of the literature.

**Methods:**

Case report and unsystematic literature review were obtained by searching the Pubmed.gov database. Thirty-six articles were identified through searches of this database and thirty-five articles were included in the selection of in-text articles integral

**Results:**

A 32-year-old men patient, without a personal or family history of psychiatric illness, was admitted to a psychiatric unit for a psychotic episode which has started three days before, mystical delusions, irritability, disorganized behavior, and aggressiveness, that had emerged shortly after a cluster of generalized tonic-clonic GTC seizures. Additionally, divided attention and memory deficits were noticed during psychiatric hospitalization.

Past medical history was relevant for epilepsy since he was 20 years olds. He did not regularly attend follow-up neurology appointments and had poor adherence to antiepileptic treatment. Last tomography images, a day before the hospitalization in psychiatry, had documented hypodense lesions in the periventricular white matter and subcortical semi-oval center distributed bilaterally and symmetrically suggestive of leukopathy. During the hospitalization, biochemical screening, renal and thyroid function were normal, serologies for B and C hepatitis were negative.

Psychotic symptoms subsided in the first 36 hours after admission upon treatment with Risperidone 4 mg/day, carbamazepine 600 mg/day, and 15O mg phenobarbital.

**Conclusions:**

From our research, we can deduce that although these syndromes are widely recognized, standard diagnostic manuals fail to acknowledge them, resulting in a noticeable lack of attention in the literature. Therefore, it is crucial for physicians to carefully examine patients with known risk factors for the symptoms of postictal psychosis.

**Disclosure of Interest:**

None Declared

